# Structural basis for a p21-activated kinase 4 and nicotinamide phosphoribosyltransferase dual inhibitor

**DOI:** 10.1107/S2059798326006145

**Published:** 2026-07-20

**Authors:** Jaehui Park, Hye Rim Hong, Sang Hyun Han, Jinsue Song, Se-Young Son, Saehae Choi, Sang Mi Park, Won-Kyu Lee, Chimari Jiko, Ji-Hun Kim, Jun-Goo Jee, Jeong Kyu Bang, Il Yeong Park, Soo Jae Lee

**Affiliations:** a College of Pharmacy Chungbuk National University Chungbuk28160 South Korea; b New Drug Development Center Osong Medical Innovation Foundation OsongCheongju Chungbuk28160 South Korea; c Clinical Research Center Masan National Tuberculosis Hospital Changwon51755 South Korea; d Division of Radiation Life Science, Institute for Integrated Radiation and Nuclear Science Kyoto University Osaka Japan; e Research Institute of Pharmaceutical Researches, College of Pharmacy Kyungpook National University Daegu41566 South Korea; f Division of Magnetic Resonance Korea Basic Science Institute (KBSI) Ochang Chungbuk28119 South Korea; Stanford University, USA

**Keywords:** PAK4, NAMPT, KPT-7523, dual inhibition, 2-aminopyridine pharmacophore

## Abstract

Simultaneous inhibition of oncogenic signaling and metabolic pathways represents a promising approach for cancer therapy. KPT-9274, a clinical stage compound, has been reported as a dual inhibitor of p21-activated kinase 4 (PAK4) and nicotinamide phosphoribosyltransferase (NAMPT), but its structural basis has remained undefined. Here, we present high-resolution crystal structures of PAK4 and NAMPT in complex with KPT-7523, an analog of KPT-9274, determined at 2.20 and 1.45 Å resolution, respectively. In PAK4, the 2-aminopyridine moiety of KPT-7523 enables dual binding, occupying the adenine-binding site for ATP and simultaneously engaging the substrate-binding cleft in the C-lobe, thereby interfering with both catalytic and regulatory functions. In NAMPT, the same scaffold inserts into the NAD^+^ active site in an extended conformation that preserves critical interactions. Biophysical assays revealed distinct affinities across the two targets. These findings highlight the 2-aminopyridine moiety as a versatile pharmacophore that is adaptable to structurally unrelated proteins and provide a framework for designing next-generation dual inhibitors in cancer therapy.

## Abstract 2

The 2-aminopyridine moiety of KPT-7523 binds p21-activated kinase 4 and nicotinamide phosphoribosyltransferase, enabling a versatile design for multi-target cancer therapy.

## 1. Introduction

Cancer continues to present one of the greatest challenges in modern medicine, driven by its remarkable complexity, adaptability and capacity to evade therapeutic intervention. Targeted therapies have transformed the oncology landscape, delivering precision and reduced toxicity. Yet, these single-target approaches often falter as tumors exploit redundant signaling networks and compensatory survival pathways to circumvent inhibition. To break this cycle, dual inhibitors capable of engaging distinct oncogenic axes simultaneously have emerged as a promising strategy for overcoming resistance mechanisms and achieving durable responses (Roy *et al.*, 2023 ▸; Stanković *et al.*, 2019 ▸; Wu *et al.*, 2022 ▸; Gu *et al.*, 2024 ▸). Among these innovative approaches, the simultaneous inhibition of p21-activated kinase 4 (PAK4) and nicotinamide phosphoribosyltransferase (NAMPT) has gained momentum due to its ability to cripple both oncogenic signaling and metabolic reprogramming, two hallmarks of cancer progression (Kudo *et al.*, 2024 ▸). PAK4, a serine/threonine kinase, regulates cytoskeletal remodeling, cell motility and survival signaling (Knaus *et al.*, 1995 ▸; Abo *et al.*, 1998 ▸; Gnesutta *et al.*, 2001 ▸). Dysregulation of PAK4 is observed in various cancers, including pancreatic, breast, lung, colorectal, liver and ovarian tumors, where it promotes proliferation, invasion, metastasis and therapy resistance (Yu *et al.*, 2022 ▸). At the molecular level, PAK4 contains both an adenine-binding site for ATP and a substrate-binding site, providing two distinct opportunities for pharmacological intervention (Aimes *et al.*, 2000 ▸; Eswaran *et al.*, 2007 ▸; Baskaran *et al.*, 2015 ▸). In parallel, NAMPT is the rate-limiting enzyme of the NAD^+^-salvage pathway and supports cancer-cell survival by maintaining intracellular NAD^+^ pools essential for energy metabolism, redox balance and DNA repair (Gasparrini & Audrito, 2022 ▸). Overexpression of NAMPT has been linked to tumor aggressiveness and therapy resistance in multiple cancer types (Shackelford *et al.*, 2013 ▸; Dalamaga *et al.*, 2018 ▸). Consequently, NAMPT inhibition has been explored as a strategy to deplete NAD^+^ levels and sensitize tumors to metabolic stress-induced apoptosis (Sampath *et al.*, 2015 ▸; Navas & Carnero, 2021 ▸).

KPT-9274 embodies this dual-targeting paradigm as a first-in-class small-molecule inhibitor of PAK4 and NAMPT. Preclinical studies have demonstrated its ability to suppress tumor progression by simultaneously disrupting oncogenic signaling and metabolic pathways (Mpilla *et al.*, 2019 ▸; Mitchell *et al.*, 2021 ▸). Based on these findings, early-phase clinical trials (NCT02702492, NCT04914845) were initiated to evaluate its safety and efficacy in patients with relapsed and refractory acute myeloid leukemia (AML), non-Hodgkin lymphoma (NHL) and solid tumors (Mogol *et al.*, 2024 ▸; Robinson *et al.*, 2024 ▸). The phase I trial (NCT02702492) was terminated following a sponsor decision based on emerging data, without public disclosure of efficacy outcomes. Meanwhile, the phase I/II trial (NCT04914845) remains active but not recruiting as of June 2024. Preclinical studies in mice reported dose-limiting toxicities, including gender-dependent nephrotoxicity and anemia, associated with NAMPT inhibition, which were partially mitigated by niacin supplementation (Mitchell *et al.*, 2021 ▸). In addition, broad NAD^+^ depletion characteristic of NAMPT inhibitors has been associated with gastrointestinal and hematopoietic toxicities (Sampath *et al.*, 2015 ▸). These observations illustrate the clinical challenges inherent to dual-targeting strategies and emphasize the need for optimization of inhibitor selectivity and binding stability.

**Figure 1:**
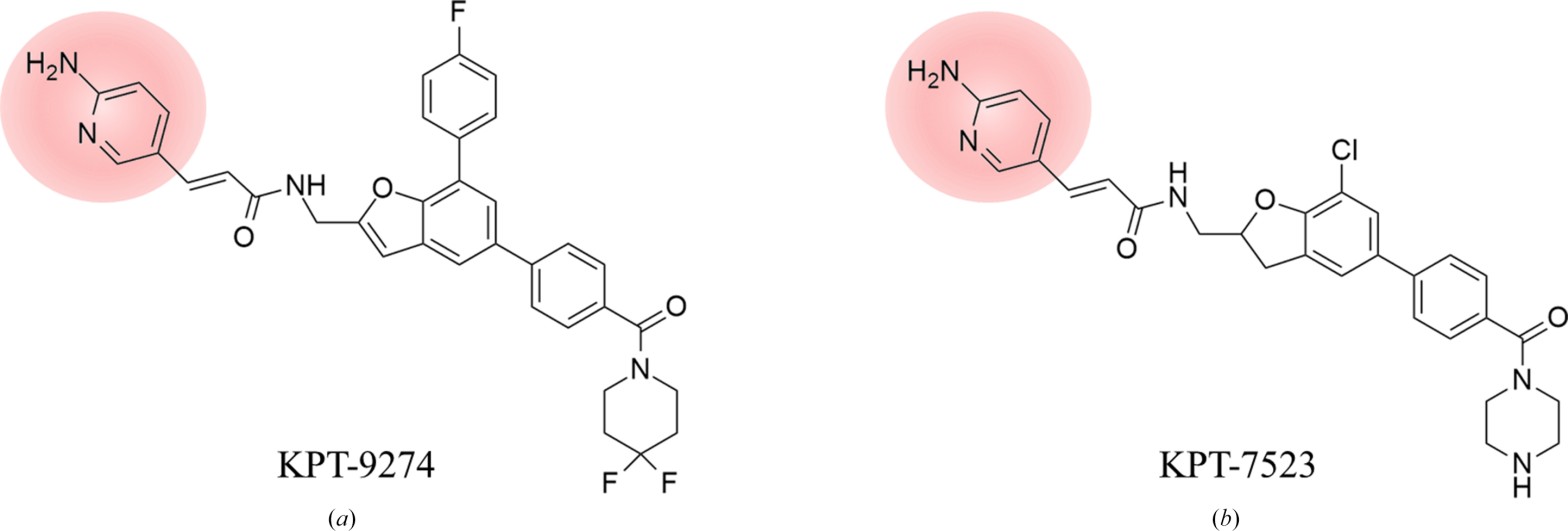
Chemical structures of (*a*) KPT-9274 and (*b*) KPT-7523. The 2-aminopyridine moieties, which are critical for dual inhibition of PAK4 and NAMPT, are highlighted in red.

Although KPT-9274 has been widely investigated as a dual PAK4/NAMPT inhibitor, the structure of its complex with PAK4 has not been reported to date. This gap in structural knowledge has limited insights into how KPT-9274 engages PAK4 at the molecular level and has hindered the rational design of improved analogs for enhanced potency and selectivity. Previous cellular studies have proposed a non-ATP-competitive mode of inhibition, in which KPT-9274 suppresses phosphorylation of PAK4 downstream effectors such as LIMK and AKT without directly binding the ATP cleft (Rane *et al.*, 2017 ▸). Functional studies further suggested that KPT-7523 and related analogs also interact with PAK4 via a similar non-ATP-competitive mechanism (Aboukameel *et al.*, 2017 ▸; Fulciniti *et al.*, 2017 ▸). However, the lack of structural data has precluded mechanistic understanding of this binding mode. To address this gap, we investigated KPT-7523, a structurally related analog of KPT-9274 featuring a more compact and conformationally restricted scaffold. KPT-7523 retains dual inhibitory activity and readily crystallized in complex with both PAK4 and NAMPT. The chemical structures of KPT-9274 and KPT-7523 highlight key substitutions that distinguish their steric and conformational profiles (Fig. 1 ▸). By resolving high-resolution co-crystal structures of KPT-7523 bound to PAK4 (2.20 Å) and NAMPT (1.45 Å), and validating them through steady-state enzyme kinetics, we demonstrate that KPT-7523 operates on PAK4 via a mixed-type inhibition mechanism rather than a purely non-ATP-competitive one. We elucidate the atomic-level interactions that enable dual inhibition, mainly via 2-aminopyridine moiety. These findings provide a structural framework for the rational design of next-generation dual inhibitors targeting oncogenic signaling and metabolic pathways.

## 2. Materials and methods

### 2.1. Cloning, expression and purification

The human PAK4 kinase domain (residues 300–591) was codon-optimized for *Escherichia coli* and cloned into the pET-28a vector. The protein was transformed in BL21(DE3) and expressed in lysogeny broth (LB) medium by induction with 0.3 m*M* isopropyl β-d-1-thiogalactopyranoside (IPTG) at an OD_600 nm_ of 0.6 for 16 h at 18°C. Purification was performed using Ni–NTA beads. The N-terminal His tag was removed for crystallization. The cleaved protein was purified by passing it through Ni–NTA resin to remove the 6×His tag and TEV protease. Subsequently, size-exclusion chromatography using a HiLoad 16/600 Superdex 75 pg column was performed with a buffer consisting of 20 m*M* HEPES pH 7.4, 150 m*M* NaCl, 1 m*M* DTT. The PAK4 protein fractions were collected and concentrated to a final concentration of 4 mg ml^−1^.

The gene encoding NAMPT was inserted into the pET-28a vector and introduced into *E. coli* strain BL21-CodonPlus(DE3) for the purpose of overexpressing the protein. The bacterial cells were cultured at 37°C until an optical density of 0.7 (OD_600 nm_) was reached. Subsequently, expression of the recombinant protein was induced by adding 0.4 m*M* IPTG and the cells were incubated for 16 h at a temperature of 20°C in LB medium. Lysates were purified with nickel Ni–NTA resin to extract the overexpressed proteins. The recombinant protein was eluted in 20 m*M* Tris–HCl pH 8.0, 200 m*M* NaCl, 0.1% Triton X-100, 5% glycerol, 150 m*M* imidazole. NAMPT was further purified using a HiLoad 16/600 Superdex 75 pg column (Cytiva) which had been pre-equilibrated with 20 m*M* Tris–HCl pH 8.0, 200 m*M* NaCl, 5% glycerol. Protein fractions were collected and concentrated to 12 mg ml^−1^.

### 2.2. Protein crystallization

KPT-7523 was synthesized by Daejung Chemicals & Metals Siheung, Korea.

The PAK4 protein (4 mg ml^−1^) was incubated with 1 m*M* KPT-7523 at room temperature for 10 min before crystallization setup. The reservoir solution consisted of 16% PEG 3350, 0.5 *M* urea, 10% MES pH 6.5, 2% phenol. The crystal belonged to space group *P*2_1_, with unit-cell parameters *a* = 88.5, *b* = 208.7, *c* = 132.5 Å, β = 103.3°. The asymmetric unit contained 12 PAK4–KPT-7523 complexes. All drops were composed of 0.25 µl protein solution and 0.25 µl reservoir solution.

The NAMPT crystals were obtained using the sitting-drop vapor-diffusion technique at a controlled temperature of 20°C. A reservoir solution composed of 22% PEG 3350 and 5% Tacsimate pH 8.0 was prepared to facilitate crystal growth. To preserve the crystals, they were cryoprotected by cooling in a solution containing 20% glycerol. The NAMPT protein (12 mg ml^−1^) was incubated with 1 m*M* KPT-7523 at room temperature for 10 min before crystallization. The crystal belonged to space group *P*2_1_, with unit-cell parameters *a* = 60.8, *b* = 107.2, *c* = 83.4 Å, β = 96.8°. The asymmetric unit contained two NAMPT–KPT-7523 complexes. The drops contained 0.5 µl each of protein and reservoir solution.

### 2.3. Data collection and processing, and structure determination

X-ray diffraction data were collected on the BL-5C beamline at the Pohang Accelerator Laboratory (PAL) located in Pohang, Korea. The collected data sets were processed using *XDS* (Kabsch, 2010 ▸). Subsequent structure refinement was conducted utilizing *Phenix* (Liebschner *et al.*, 2019 ▸), while model building was performed using *Coot* (Emsley & Cowtan, 2004 ▸). Crystallographic restraints for the ligand KPT-7523 were generated from its SMILES string using the *electronic Ligand Builder and Optimization Workbench* (*eLBOW*) module within the *Phenix* suite, employing the AM1 semi-empirical quantum-mechanical method for geometry optimization. For the presentation of detailed data and refinement statistics, please refer to Table 1 ▸ (PDB entries 9wdf and 9wdd). All structural figures depicted in this paper were generated using *PyMOL* (version 1.8; Schrödinger).

### 2.4. *In vitro* kinase activity and steady-state kinetics assays

The catalytic activity and steady-state kinetics of PAK4 were evaluated using the ADP-Glo Kinase Assay kit (Promega, Madison, Wisconsin, USA). The kinase reactions were carried out in 96-well plates in an assay buffer consisting of 40 m*M* Tris–HCl pH 7.5, 5 m*M* MgCl_2_, 5% glycerol, 1 m*M* DTT, 0.1 mg ml^−1^ BSA. For the determination of apparent Michaelis–Menten constant (*K*_m_) values and the mode of inhibition, purified recombinant PAK4 was used at a fixed amount of 3 ng per well. To determine the mechanism of KPT-7523 inhibition via ATP titration, the concentration of the substrate peptide (PAKtide; sequence RRRLSFAEPG) was fixed at a saturating concentration of 100 µ*M*, while ATP concentrations were varied from 50 to 0.04 µ*M* using twofold serial dilutions. KPT-7523 was added at fixed concentrations of 0, 5 and 30 µ*M*. Reactions were initiated by the addition of the ATP and substrate mixture, yielding an initial reaction volume of 10 µl. The plates were incubated at 25°C for 20 min to ensure the reactions proceeded within the steady-state linear range. Following the kinase reaction, 10 µl ADP-Glo Reagent was added to terminate the reaction and deplete unconsumed ATP. After a 40 min incubation at 25°C, 20 µl kinase detection reagent was added to generate a luminescent signal proportional to the ADP produced. Following an additional 20 min incubation, the luminescence was recorded using a Spectra­Max iD3 microplate reader.

An ADP standard curve was generated under identical assay conditions to correlate the raw relative light units (RLU) to the absolute concentration of ADP produced. The initial reaction velocities (*V*_0_) were plotted against ATP concentrations, and the apparent *K*_m_ and maximum velocity (*V*_max_) values were calculated through nonlinear regression analysis using the Michaelis–Menten equation in *GraphPad Prism* 8.0. The mode of inhibition was further evaluated by transforming the data into Lineweaver–Burk double-reciprocal plots. All assays were performed in duplicate, and the data are presented as the mean ± standard deviation (SD).

### 2.5. Microscale thermophoresis binding assays

His_6_-PAK4 and His_6_-NAMPT were diluted to 200 n*M* in assay buffer (20 m*M* Tris pH 7.5, 150 m*M* NaCl, 5% glycerol, 1 m*M* DTT, 0.1% PEG 8000, 0.05% Tween-20). RED-tris-NTA dye was diluted in the same buffer to a final concentration of 100 n*M*. The proteins (100 µl) were incubated with 100 µl dye solution for 30 min at room temperature in the dark. Samples were centrifuged at 15 000*g* for 10 min at 4°C and the supernatants were transferred to fresh tubes. KPT-7523 was stored as a 100% DMSO stock at −20°C. For the binding assay, a 1 m*M* intermediate stock of KPT-7523 was prepared in assay buffer (final DMSO concentration 5%) and used to generate a 16-point serial dilution in the same buffer. Each dilution (10 µl) was mixed with 10 µl 200 n*M* RED-labeled protein (PAK4 or NAMPT, respectively), followed by gentle mixing by pipetting. After incubation for 10 min at room temperature protected from light, samples were loaded into Monolith NT.115 Premium capillaries (NanoTemper Technologies). To clarify, the binding events were monitored exclusively through the fluorescence signal originating from the site-specific RED-tris-NTA dye attached to the His-tag of the target proteins, rather than intrinsic protein tryptophan fluorescence. MST measurements were performed using a NanoTemper RED detector, with the dye’s fluorescence excitation and emission maxima at approximately 650 and 670 nm, respectively. The analysis was carried out at 25°C using 39% LED power (RED channel) and medium MST power for PAK4, and 38% LED power and medium MST power for NAMPT. Dissociation constants (*K*_d_) were determined using the *MO.Affinity Analysis* software (NanoTemper Technologies). Notably, MST measurements for KPT-9274 could not be performed under these assay conditions due to its limited aqueous solubility, which precluded reliable biophysical characterization.

## 3. Results

### 3.1. Crystal structure of the PAK4–KPT-7523 complex

**Figure 2:**
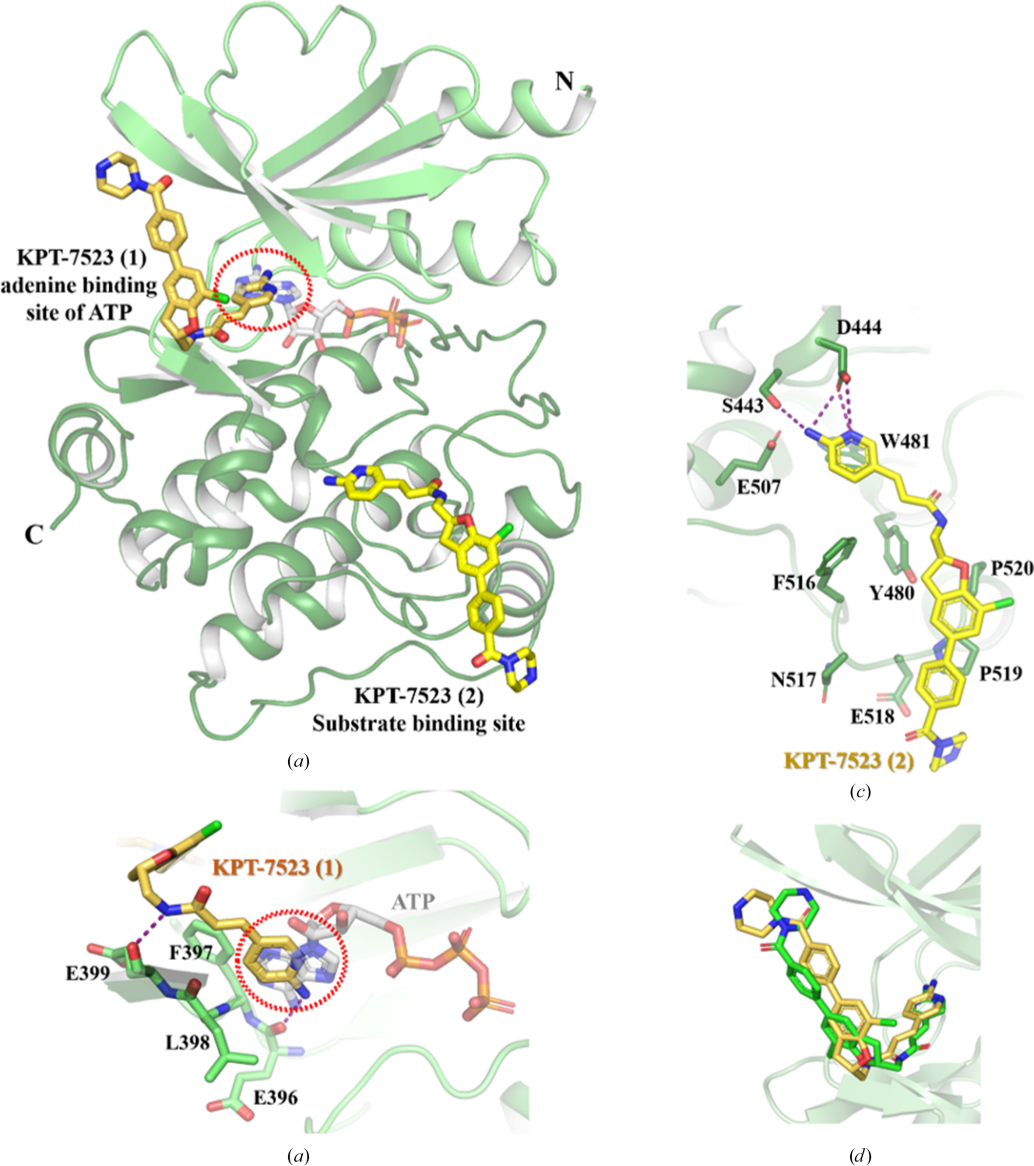
Crystal structure of the PAK4 kinase domain in complex with KPT-7523. (*a*) Overall structure of PAK4 showing KPT-7523 bound at two distinct sites: (1) the adenine-binding site of ATP (ligand shown in yellow–orange) within the N-lobe (lime) and (2) a substrate-binding site (ligand shown in yellow) within the C-lobe (forest). The N- and C-terminal regions are labeled for orientation. For reference, an ATP molecule from a previously reported structure (PDB entry 4xbr) is overlaid in silver. (*b*) Close-up view of the adenine-binding site of ATP showing KPT-7523 (yellow–orange) stabilized by hydrogen bonds (purple dashed lines) and hydrophobic interactions. (*c*) Close-up view of the substrate-binding site with KPT-7523 (yellow) engaged in key hydrogen-bonding and hydrophobic contacts with surrounding residues. (*d*) Structural superposition of the *Boltz*-2-generated complex model (green) onto the experimental crystal structure (yellow–orange). The computational model accurately reproduces the experimentally observed KPT-7523 pose at the entry of the ATP-binding cleft (see the supporting information for details).

The inhibitory effects of KPT-9274 on PAK4, as reported in multiple studies, prompted us to investigate its molecular interactions with PAK4 through co-crystallization experiments. Despite repeated attempts, electron density corresponding to KPT-9274 was not observed in the diffraction data, preventing structural characterization of its interactions with PAK4. To overcome this limitation, we employed KPT-7523, a structurally related analog of KPT-9274 with reduced steric bulk and enhanced rigidity (Aboukameel *et al.*, 2017 ▸; Naïja *et al.*, 2021 ▸). This compact scaffold enabled successful crystallization and provided structural insights into simultaneous engagement of the catalytic and substrate-binding sites of PAK4. The crystal structure of the human PAK4 kinase domain bound to KPT-7523 was determined at 2.20 Å resolution, revealing dual occupancy of two distinct functional sites: the adenine-binding site for ATP in the N-lobe and the substrate-binding pocket in the C-lobe (Fig. 2 ▸*a*; Eswaran *et al.*, 2007 ▸). The asymmetric unit of the PAK4–KPT-7523 complex contained 12 PAK4 molecules, each bound to two KPT-7523 molecules. Structural alignment showed high similarity across all chains (average r.m.s.d. 0.54 Å), consistently supporting the dual-binding mode. Importantly, this structural observation provides a mechanistic explanation for the previously reported stoichiometry of 2 in isothermal titration calorimetry (ITC) experiments (Fulciniti *et al.*, 2017 ▸), in which two KPT-7523 molecules were shown to bind per PAK4 monomer. These data together suggest that KPT-7523 engages PAK4 through a dual mechanism that interferes with both catalytic and regulatory functions.

**Figure 3:**
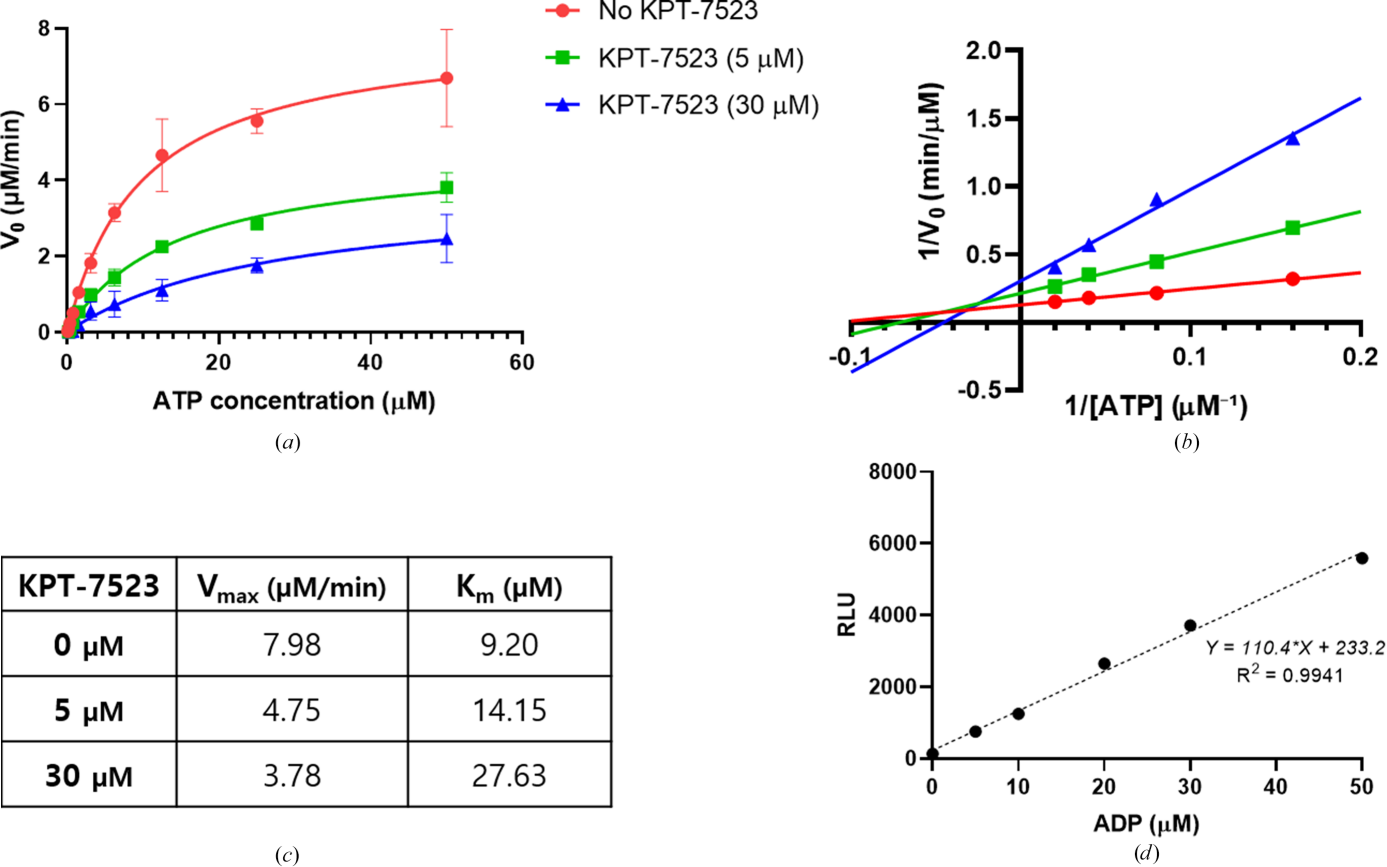
Steady-state kinetic analysis of PAK4 inhibition by KPT-7523. (*a*) Michaelis–Menten plot of initial PAK4 reaction velocities (*V*_0_) versus ATP concentrations. Data are presented as mean ± SD of replicates. (*b*) Lineweaver–Burk double-reciprocal plot of the data from (*a*). (*c*) Kinetic parameters (*V*_max_ and *K*_m_) derived from nonlinear regression analysis of the Michaelis–Menten curves in (*a*). (*d*) Standard curve for ADP quantification (*R*^2^ = 0.9941).

At the adenine-binding site of ATP, KPT-7523 overlaps the adenine-ring position of ATP (Figs. 2 ▸*a* and 2 ▸*b*, red circle), as observed in the reference structure (PDB entry 4xbr). The amino group of the 2-aminopyridine moiety forms a hydrogen bond to the side-chain O atom of Glu396, anchoring the core of the ligand deep within the ATP cleft. Further along the extended scaffold, an amide N atom forms an additional hydrogen bond to the backbone carbonyl of Glu399. Supported by hydrophobic contacts involving Leu346, Phe397 and Leu398, this deep hydrophobic pocket cradles the 2-aminopyridine ring of KPT-7523, stabilizing its binding conformation (Fig. 2 ▸*b*). Importantly, this deep insertion of the aminopyridine ring effectively occludes ATP access (Fig. 2 ▸*a*), supporting the competitive inhibition mechanism demonstrated in our steady-state kinetics (Figs. 3 ▸*a* and 3*b* ▸). The solvent-exposed portion of KPT-7523 that extends outside the ATP site forms intermolecular contacts with another KPT-7523 from a neighboring PAK4 monomer, resulting in a *C*2 dimer configuration that stabilizes ligand binding within the crystal packing. At the substrate-binding site in the C-lobe (Fig. 2 ▸*c*), KPT-7523 engages a substrate-binding pocket distinct from the catalytic site. The two N atoms of the 2-aminopyridine moiety form hydrogen bonds to the side chains of Ser443 and Asp444, anchoring the ligand within the groove. A π–π stacking interaction between the 2-aminopyridine ring of KPT-7523 and the indole ring of Trp481 (distance of ∼3.8 Å, angle of ∼90°) provides additional stabilization. Notably, this pocket corresponds to a site identified in our X-ray fragment-based ligand discovery (X-FBLD) studies (unpublished data), supporting its potential as a platform for allosteric inhibitor development. Hydrophobic interactions (Trp481, Phe516, Pro519 and Pro520) form a tightly packed environment that reinforces KPT-7523 anchoring, which is further stabilized by interactions with the main-chain atoms of Asn517 and Glu518, as well as the side chain of Tyr480 (Fig. 2 ▸*c*). Furthermore, close intermolecular contacts from Tyr320, Val340 and Lys345 of the neighboring PAK4 molecule contribute to the overall stabilization of the ligand. These findings reveal that KPT-7523 simultaneously targets two distinct sites within PAK4 (Fig. 2 ▸*a*), providing high-resolution structural insights into a dual-binding mechanism that interferes with both ATP turnover and substrate engagement. This represents rare crystallographic evidence of a small molecule occupying both the catalytic ATP site and the substrate pocket of a kinase. This dual occupancy establishes a novel paradigm for kinase inhibition and offers a blueprint for the design of next-generation bifunctional inhibitors that can modulate multiple functional domains within their targets.

### 3.2. Steady-state kinetics reveal a mixed-type inhibition of PAK4 by KPT-7523

To validate the dual-site binding mode of KPT-7523 observed in our crystal structures, we performed steady-state enzyme kinetics. We measured the initial velocity (*V*_0_) of the PAK4 kinase reaction across varying concentrations of ATP in the presence of KPT-7523 (0, 5 and 30 µ*M*). As shown by the Michaelis–Menten saturation curves, KPT-7523 dose-dependently suppressed the kinase activity of PAK4 (Fig. 3 ▸*a*). Kinetic analysis revealed that increasing KPT-7523 concentrations increased the apparent Michaelis constant (*K*_m_) from 9.20 to 27.63 µ*M*, indicating a reduction in ATP binding affinity consistent with a competitive mechanism (Fig. 3 ▸*c*). Concurrently, the maximum reaction velocity (*V*_max_) decreased from 7.98 to 3.78 µ*M* min^−1^, reflecting an impairment of the enzyme’s catalytic turnover characteristic of a non­competitive interaction. To definitively determine the mode of inhibition, the data were transformed into a Lineweaver–Burk double-reciprocal plot (Fig. 3 ▸*b*). The resulting plot exhibited a classical mixed-type inhibition pattern (Klemm *et al.*, 2022 ▸). These biochemical findings revise the previously proposed purely non-ATP-competitive mechanism and corroborate our structural model, confirming that KPT-7523 acts as a dual-site inhibitor by occupying both the ATP-binding pocket and the substrate-binding cleft (Fig. 2 ▸*a*).

### 3.3. Computational characterization of PAK4 kinase domain–small molecule interactions

To characterize PAK4–ligand interactions computationally, we applied three AI-based co-folding methods [*AlphaFold*3 (Abramson *et al.*, 2024 ▸), *Boltz*-2 (Passaro *et al.*, 2025 ▸) and *Protenix* (ByteDance AML AI4Science Team *et al.*, 2025 ▸)] and two ligand-binding-site prediction methods [*FTMap* (Kozakov *et al.*, 2015 ▸) and *AF*2*BIND* (Gazizov *et al.*, 2023 ▸)], using the PAK4 kinase-domain sequence (UniProt O96013, residues 300–591) and the ligand SMILES as inputs. The apo co-folded model agreed closely with the experimental holo structure (C^α^ r.m.s.d. 0.92 Å), but only *Boltz*-2 reproduced the experimentally observed pose at the entry to the ATP-binding cleft (Fig. 2 ▸*d*); *AlphaFold*3 and *Protenix* placed the ligand deeper in the ATP pocket in a type-II-like mode, and none of the three predicted the secondary, allosteric site (Supplementary Fig. S1). To localize the allosteric site without reference to the crystallographic ligand position, we applied *FTMap* and *AF*2*BIND*. The eighth *FTMap* consensus cluster and an *AF*2*BIND* residue cluster — Asp444 at high confidence [*P*(bind) = 0.833] with three flanking residues at moderate confidence (Thr404, 0.521; Lys442, 0.631; Thr478, 0.545) — coincided spatially on the C-lobe surface with the binding site of the aminopyridyl acrylamide warhead of the second ligand (Supplementary Fig. S2). Because both methods are blind to the crystallographic ligand position, their convergence supports the assignment of this site as a genuine surface pocket. The aminopyridyl acrylamide is the SAR-defined pharmacophore of the KPT-9274 chemotype (Li *et al.*, 2025 ▸), and its localization at this independently predicted pocket suggests that the second binding site may correspond to the allosteric binding region of this chemotype, whose structural location has not previously been resolved.

### 3.4. Crystal structure of the NAMPT–KPT-7523 complex

**Figure 4:**
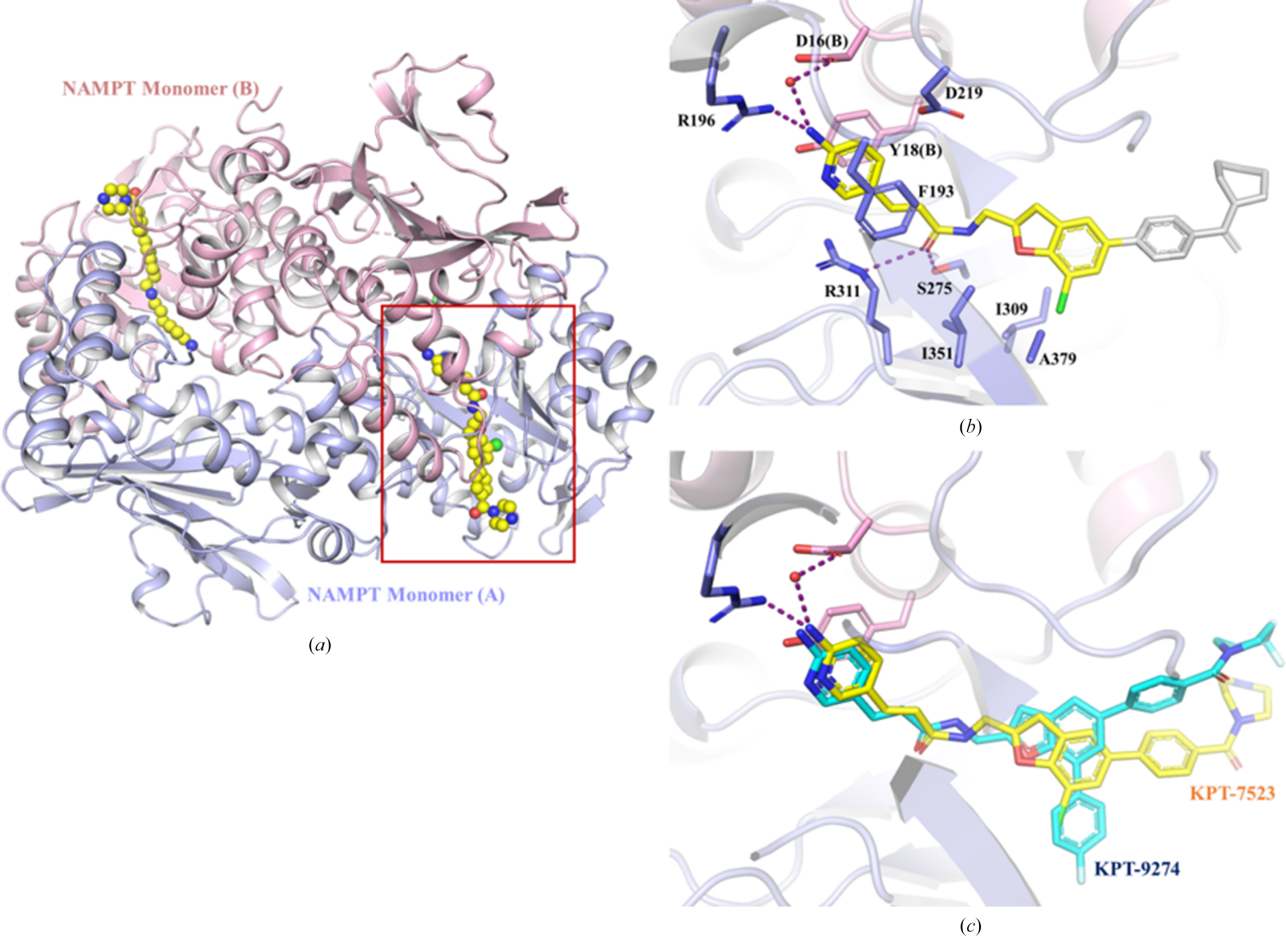
Co-crystal structure of the NAMPT dimer with KPT-7523. (*a*) The crystal structure of the NAMPT dimer with KPT-7523 is depicted as a cartoon with the NAMPT monomers shown in light blue (*A* chain) and light pink (*B* chain) and KPT-7523 in yellow ball-and-stick representation. (*b*) Details of KPT-7523 (yellow) binding (a fragment with unclear electron density is depicted in gray). Important hydrogen bonds are depicted as deep purple dashed lines. (*c*) Structural overlay of KPT-7523 (yellow) and KPT-9274 (cyan) bound to NAMPT (PDB entry 5nsd), highlighting their distinct binding conformations.

The crystal structure of NAMPT in complex with KPT-7523 was determined at 1.45 Å resolution, revealing a homodimer in the asymmetric unit, with each monomer binding one molecule of KPT-7523 (Fig. 4 ▸*a*). Within the NAD^+^-binding pocket, KPT-7523 adopts an extended and well defined conformation that anchors the ligand through multiple stabilizing interactions across the dimer interface. This binding mode closely mirrors that of KPT-9274 as previously reported (PDB entry 5nsd; Neggers *et al.*, 2018 ▸). A key feature shared by both inhibitors is the 2-aminopyridine moiety, which forms a water-mediated hydrogen bond to Asp16 (chain *B*) and a direct hydrogen bond to Arg196 (chain *A*), anchoring the ligand within the core of the active site (Fig. 4 ▸*b*). Further stabilization is provided by a hydrogen bond to Ser275 (chain *A*), as well as hydrophobic contacts involving Ile309, Ile351 and Ala379 (all chain *A*), creating a compact, hydrophobically stabilized pocket around the ligand. Notably, KPT-7523 forms an additional hydrogen bond to Arg311 (chain *A*) that is absent in the KPT-9274 complex. This interaction likely restricts the orientation of the ligand near the entrance to the active site, contributing to its more rigid and conformationally stable pose.

**Figure 5:**
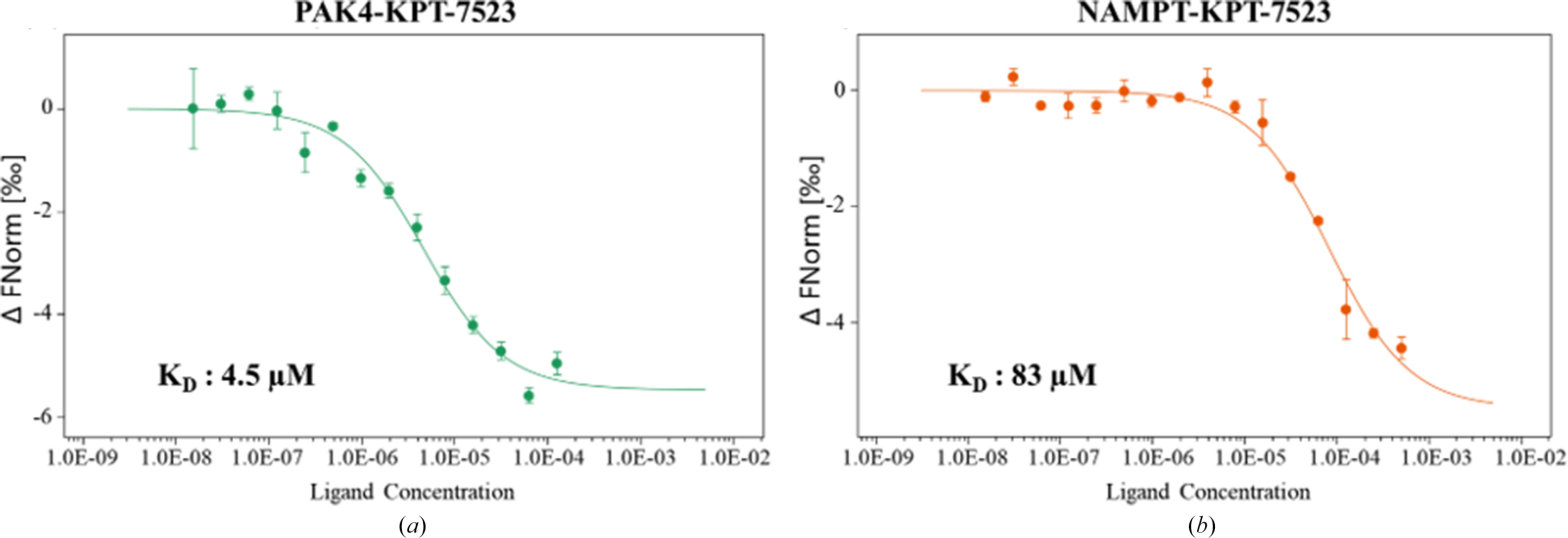
Dose–response curves for KPT-7523 binding to His-tagged PAK4 (*a*) and NAMPT (*b*) were obtained using RED-tris-NTA-labeled proteins. Error bars represent the standard deviation from three independent experiments. FNorm, normalized fluorescence.

To further compare the surface-interaction profiles of KPT-7523 and KPT-9274, solvent-accessible surface area (SASA) analysis was performed. KPT-7523 exhibited an average SASA of 462 Å^2^ per monomer, while KPT-9274 showed a larger value of 523 Å^2^. This difference suggests that KPT-9274 interacts with a broader and more solvent-exposed protein interface, consistent with its flexible fluorinated piperidine moiety (Fig. 4 ▸*c*). In contrast, KPT-7523 relies on a more compact and geometrically constrained scaffold that localizes binding contacts to a smaller interface. While this may reduce the overall binding surface area, it likely enhances interaction specificity and structural rigidity within the active site. Although KPT-7523 exhibits lower binding affinity for NAMPT (*K*_d_ = 83 µ*M*; Fig. 5 ▸*b*), the crystallographic evidence demonstrates a reproducible and well ordered binding pose that closely resembles that of KPT-9274. This structural congruence demonstrates that KPT-7523 serves as a valid structural surrogate for capturing the key molecular inter­actions within the NAMPT active site.

### 3.5. Differential binding affinities of KPT-7523 to PAK4 and NAMPT as determined by MST

MST measurements revealed distinct binding affinities of KPT-7523 for PAK4 and NAMPT, reflecting differential interactions with these two oncogenic targets (Fig. 5 ▸). The MST-derived dissociation constant (*K*_d_) for PAK4 was 4.5 µ*M*, which aligns closely with previously reported ITC data (*K*_d_ = 3.7 µ*M*; Fulciniti *et al.*, 2017 ▸). In contrast, the weaker affinity for NAMPT (*K*_d_ = 83 µ*M*) reflects its monomer-level engagement at the NAD^+^-binding site, as observed in the NAMPT–KPT-7523 crystal structure, where each monomer binds one ligand. Because the dual binding events in PAK4 could not be distinctly resolved under our experimental conditions, MST dose–response curves were fitted to a standard one-site binding model to derive an overall macroscopic *K*_d_ (Tso *et al.*, 2018 ▸). *K*_d_ values represent the mean ± standard deviation of three independent experiments, each performed with technical triplicates. All MST measurements were carried out at 25°C, using either high or medium MST power settings. The LED power was set to 39% for PAK4 titrations and to 38% for NAMPT titrations. These MST measurement results provide quantitative evidence for the bifunctional inhibitory profile of KPT-7523 and support its differential binding affinities across oncogenic kinase and metabolic targets.

## 4. Discussion

While KPT-9274 has been extensively studied in preclinical and clinical contexts as a dual inhibitor of PAK4 and NAMPT (Mpilla *et al.*, 2019 ▸; Neggers *et al.*, 2018 ▸; Fulciniti *et al.*, 2017 ▸), the precise structural basis for its activity towards PAK4 has remained elusive. Several studies have described KPT-9274 as an allosteric modulator of PAK4, based on its ability to suppress downstream signaling without competing for ATP binding (Abu Aboud *et al.*, 2016 ▸; Aboukameel *et al.*, 2017 ▸; Fulciniti *et al.*, 2017 ▸; Rane *et al.*, 2017 ▸). These conclusions were primarily drawn from cellular assays reporting reduced phosphorylation of PAK4 downstream targets, such as LIMK, β-catenin and AKT. However, to date, no direct structural or biophysical evidence has confirmed the binding of KPT-9274 to PAK4. Moreover, the possibility of pleiotropic effects and off-target interactions has been raised in several studies (Rane *et al.*, 2017 ▸), further complicating mechanistic interpretation and underscoring the need for structural clarity. To address these uncertainties, we determined the crystal structure of PAK4 in complex with KPT-7523, a structurally related analog of KPT-9274 (Fig. 2 ▸*a*).

The crystal structure of PAK4 bound to KPT-7523 reveals a dual engagement mode in which the inhibitor simultaneously occupies the adenine-binding site of ATP in the N-lobe and the substrate-binding site in the C-lobe (Fig. 2 ▸*a*). This dual-site occupancy is stabilized by a network of six hydrogen bonds, two in the ATP-binding site (Glu396 and Glu399) and four in the substrate-binding site (Ser443 and Asp444), along with π–π stacking with Trp481 and extensive hydrophobic interactions (Figs. 2 ▸*b* and 2 ▸*c*). This dual-site binding mode aligns with previously reported ITC data indicating a 2:1 stoichiometry between KPT-7523 and PAK4 (Fulciniti *et al.*, 2017 ▸), supporting the notion that both the catalytic and regulatory surfaces are targeted. Although MST analysis yielded a single macroscopic *K*_d_ due to the convolution of these multiple binding events, our unambiguous crystallographic data physically substantiate this 2:1 engagement. Consistent with this structural model, steady-state enzyme kinetics confirmed that KPT-7523 functionally impairs PAK4 activity by simultaneously occupying both the ATP-binding pocket and the substrate-binding cleft (Figs. 3 ▸*a* and 3 ▸*b*). The observed concurrent increase in *K*_m_ and decrease in *V*_max_ functionally corroborate the dual-site structural mode, demonstrating that KPT-7523 effectively disrupts both substrate turnover and ATP engagement.

In parallel, structural characterization of NAMPT in complex with KPT-7523 revealed a binding conformation closely resembling that of KPT-9274, including conserved interactions at the NAD^+^-binding site. The crystallographic data show a rigid and reproducible binding mode, suggesting high conformational stability. SASA analysis revealed a more localized protein–ligand interface compared with KPT-9274, reflecting the compact scaffold of KPT-7523. For NAMPT, MST measurements established a biophysical binding affinity (*K*_d_) of 83 µ*M* for KTP-7523. While KPT-9274 is well documented as a potent functional inhibitor (Abu Aboud *et al.*, 2016 ▸), its higher potency likely stems from extended surface interactions facilitated by its flexible terminal moieties. Crucially, the core pharmacophoric determinants required for target engagement, specifically mediated by the 2-amino­pyridine moiety, remain entirely conserved within KPT-7523. Therefore, rather than focusing on the disparity between absolute kinetic values, we emphasize that KPT-7523 serves as a high-fidelity structural probe that precisely captures the essential binding mechanism. This structural congruence confirms that KPT-7523 successfully preserves the pharmacophoric core of dual inhibition, providing an essential structural blueprint to inform future optimization strategies.

Together, these data establish KPT-7523 as a dual-binding probe that enables structural elucidation of both PAK4 and NAMPT inhibition. The identification of a dual-occupancy mode in PAK4, targeting both the adenine-binding site of ATP in the N-lobe and the substrate-binding site in the C-lobe, and its biochemical validation as a mixed-type inhibitor provides novel mechanistic insight into kinase inhibition beyond classical models. A key determinant of the dual-targeting capability of KPT-7523 is the 2-aminopyridine moiety, which mediates conserved hydrogen bonds in both PAK4 and NAMPT despite their divergent active-site architectures. This pharmacophore acts as a versatile anchor, facilitating geometric adaptability and precise engagement within two chemically distinct environments. These findings underscore the importance of such adaptable motifs in enabling simultaneous inhibition of unrelated oncogenic pathways. Building on these structural and biophysical insights, future optimization efforts could focus on libraries of 2-aminopyridine-containing compounds, using computational docking to identify scaffolds with improved selectivity and potency. This structure-guided approach establishes a mechanistic blueprint for rational dual-inhibitor design, illustrating how strategic pharmacophore placement can be exploited to develop next-generation therapeutics that simultaneously disrupt orthogonal oncogenic pathways.

## Supplementary Material

supplementary. DOI: 10.1107/S2059798326006145/wa5155sup1.docx

PDB reference: NAMPT–KPT-7523 complex, 9wdd

PDB reference: PAK4–KPT-7523 complex, 9wdf

## Figures and Tables

**Table 1:** Data collection and processing, and structure determination Values in parentheses are for the highest resolution shell.

	PAK4–KPT-7523	NAMPT–KPT-7523
Data collection		
Space group	*P*12_1_1	*P*12_1_1
Wavelength (Å)	1.0	1.0
Temperature (K)	100	100
*a*, *b*, *c* (Å)	88.5, 208.7, 132.5	60.8, 107.2, 83.4
α, β, γ (°)	90.0, 103.3, 90.0	90.0, 96.8, 90.0
Resolution (Å)	44.76–2.20 (2.23–2.20)	29.53–1.45 (1.47–1.45)
*R*_merge_	0.051 (0.607)	0.072 (0.558)
〈*I*/σ(*I*)〉	9.6 (1.2)	5.4 (1.0)
Completeness (%)	96.56 (90.0)	89.19 (35.0)
Refinement		
Resolution (Å)	2.20	1.80
No. of reflections	452526	331037
*R*_work_/*R*_free_	0.22/0.25 (0.37/0.37)	0.18/0.19 (0.43/0.38)
No. of atoms		
Protein	27404	7508
Water	163	1088
*B* factors (Å^2^)		
Overall	61.4	23.9
Protein	33.6	27.5
Water	47.6	37.8
R.m.s. deviations		
Bond lengths (Å)	0.011	0.010
Bond angles (°)	1.48	1.35
Ramachandran plot (%)		
Favored	94.92	97.20
Generously allowed	3.67	2.80
PDB code	9wdf	9wdd

## Data Availability

All data needed to evaluate the conclusions in the paper are present in the paper.
