# Supplementary material for: Structural basis for a p21-activated kinase 4 and nicotinamide phosphoribosyltransferase dual inhibitor
Source: Acta Crystallogr D Struct Biol. 2026 Jul 20;82(Pt 8):972–81. doi: 10.1107/S2059798326006145 (PMC13431643; doi:10.1107/S2059798326006145)
Supplement: Supplementary file 1 [file d-82-00972-sup1.docx]

**Computational characterization of PAK4 kinase domain – small molecule interactions**

To characterize the interactions between the PAK4 kinase domain and the small molecule, we applied five complementary computational approaches: three AI-based protein–ligand co-folding methods (AlphaFold3, (Abramson *et al.*, 2024); Boltz-2, (Passaro *et al.*, 2025); Protenix, (Team *et al.*, 2025)) and two ligand-binding site prediction methods (FTMap, (Kozakov *et al.*, 2015); AF2BIND, (Gazizov *et al.*, 2023)). All co-folding runs were performed according to the developers’ recommended protocols, using the PAK4 kinase domain sequence (UniProt O96013, residues 300–591) and KPT-7523’s SMILES as inputs.

The apo PAK4 model generated by the co-folding methods was nearly indistinguishable from the experimentally determined holo structure, with a Cα RMSD of 0.92 Å across the kinase domain. Despite this close agreement at the apo level, the three methods produced markedly different complex models (Fig. S1). Only Boltz-2 reproduced the experimentally observed pose at the entry of the ATP-binding cleft (Fig. S1); AlphaFold3 and Protenix instead placed the ligand deeper into the ATP pocket, with the molecule extending toward the DFG region in a manner reminiscent of type-II kinase inhibitors. Because Boltz-2 had no access to the crystal structure during inference, its independent recovery of this pose provides additional support for the crystallographic interpretation of the primary binding site. None of the three methods, however, predicted a ligand pose at the secondary, allosteric site observed in the crystal structure. Attempts to co-fold the protein with 1:2 protein:ligand stoichiometry did not produce physically reasonable models, with significant atomic overlap between the two ligand copies, consistent with the recognized limitation that current AI-based co-folding methods are not yet optimized for multi-site ligand occupancy.

To identify candidate binding pockets independently of the crystallographically observed ligand positions, we then applied FTMap and AF2BIND. FTMap (Kozakov *et al.*, 2015) is a publicly available server that maps small-molecule binding hot spots on a protein surface by docking sixteen small organic probes (including ethanol, acetonitrile, acetone, benzene, and urea) across the protein surface, clustering the favorably bound probes by energy, and ranking the resulting consensus sites by probe occupancy. AF2BIND (Gazizov *et al.*, 2023) is a logistic regression model trained on the internal pair representation of AlphaFold2 that predicts, for each residue of a target protein, the probability of contacting a small-molecule ligand. The method requires no multiple sequence alignment, homology template, or prior ligand information; instead, it uses twenty amino-acid “bait” features to score the probability that each target residue contacts a ligand. AF2BIND has been shown to recover approximately two-thirds of known allosteric sites in benchmark sets of human proteins from AlphaFold2-predicted structures alone, indicating that it is particularly well-suited to identifying allosteric and non-conserved pockets.

We examined predicted sites lying outside the canonical ATP-binding pocket targeted by type-I and type-II kinase inhibitors. The 8th consensus cluster from FTMap and a cluster of AF2BIND residues — Asp-444, identified with high binding probability (P_bind_ = 0.833), together with three flanking residues at moderate confidence (Thr-404, P_bind_ = 0.521; Lys-442, 0.631; Thr-478, 0.545) — coincided spatially with the same region on the C-lobe surface (Fig. S2). This region corresponds to the binding site of the aminopyridyl acrylamide warhead of the allosteric ligand molecule in the crystal structure (Fig. S2). Because FTMap and AF2BIND operate without any knowledge of the crystallographic ligand position, their convergence on this site provides independent computational support that the second binding site is a genuine surface pocket on PAK4 rather than an incidental feature of the lattice. The aminopyridyl acrylamide is the SAR-defined pharmacophore of the KPT-9274 chemotype (Li *et al.*, 2025), and its localization at this independently predicted pocket suggests that the second binding site may correspond to the allosteric binding region of the KPT-9274 chemotype, whose structural location has not previously been resolved. Together with the crystallographic evidence presented above, these computational predictions support the assignment of the second binding site as a genuine ligand-binding pocket on PAK4.


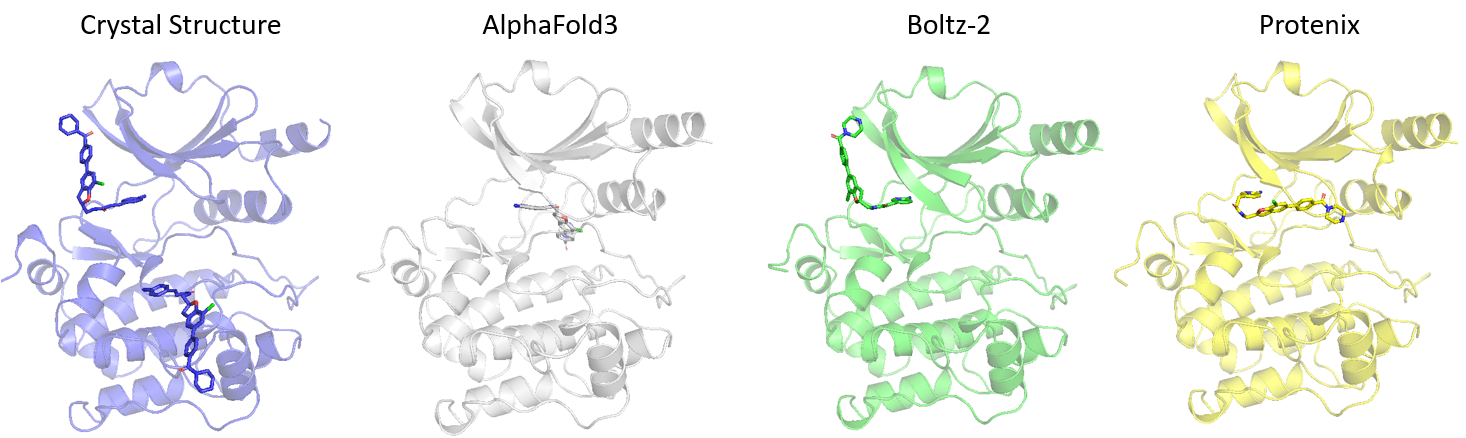


**Fig. S1**. Comparison of AI-based protein–ligand co-folding predictions for the PAK4 kinase domain (UniProt O96013, residues 300–591) with the experimentally determined holo structure. Complex models generated by AlphaFold3 (Abramson *et al.*, 2024), Boltz-2 (Passaro *et al.*, 2025), and Protenix (Team *et al.*, 2025) are superposed on the crystal structure (Cα RMSD of the apo model = 0.92 Å across the kinase domain). Only Boltz-2 reproduces the experimentally observed ligand pose at the entry of the ATP-binding cleft; AlphaFold3 and Protenix place the ligand deeper into the ATP pocket, with the molecule extending toward the DFG region in a manner reminiscent of type-II kinase inhibitors. None of the three methods predicts a ligand pose at the secondary, allosteric site observed in the crystal structure.


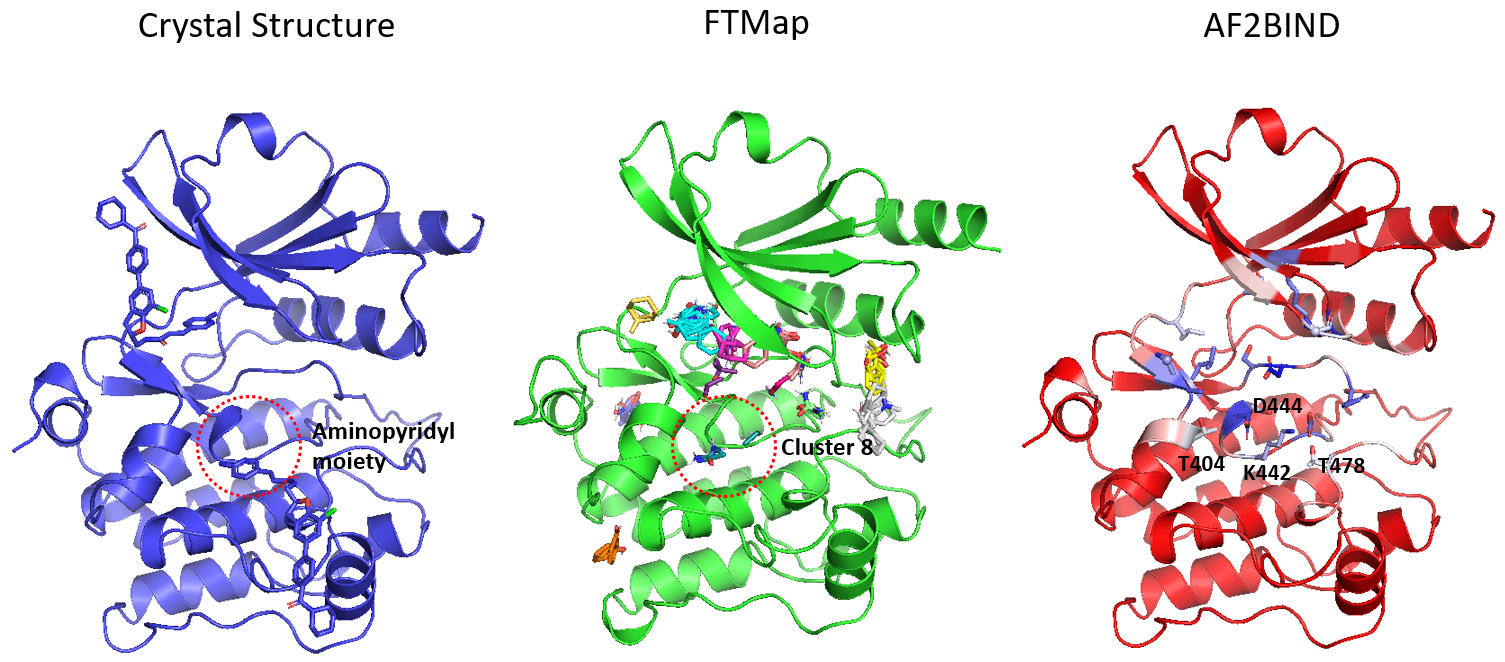


**Fig. S2.** Independent computational support for the secondary, allosteric binding site on PAK4 from FTMap (Kozakov *et al.*, 2015) and AF2BIND(Gazizov *et al.*, 2023). The 8th consensus cluster from FTMap and a cluster of AF2BIND residues — Asp-444 (Pbind = 0.833) together with three flanking residues at moderate confidence (Thr-404, Pbind = 0.521; Lys-442, 0.631; Thr-478, 0.545) — coincide spatially on the C-lobe surface, outside the canonical ATP-binding pocket. This region corresponds to the binding site of the aminopyridyl acrylamide warhead of the allosteric ligand observed in the crystal structure. Because both methods operate without knowledge of the crystallographic ligand position, their convergence on this site supports the assignment of the second binding site as a genuine surface pocket on PAK4.

**Supplementary References**

Abramson, J., Adler, J., Dunger, J., Evans, R., Green, T., Pritzel, A., Ronneberger, O., Willmore, L., Ballard, A. J. & Bambrick, J. (2024). *Nature* **630**, 493-500.

Gazizov, A., Lian, A., Goverde, C., Ovchinnikov, S. & Polizzi, N. F. (2023). *bioRxiv*, 2023.2010. 2015.562410.

Kozakov, D., Grove, L. E., Hall, D. R., Bohnuud, T., Mottarella, S. E., Luo, L., Xia, B., Beglov, D. & Vajda, S. (2015). *Nature protocols* **10**, 733-755.

Li, Y., Fang, Y., Chen, X., Tong, L., Feng, F., Zhou, Q., Chen, S., Ding, J., Xie, H. & Zhang, A. (2025). *Acta Pharmaceutica Sinica B* **15**, 438-466.

Passaro, S., Corso, G., Wohlwend, J., Reveiz, M., Thaler, S., Somnath, V. R., Getz, N., Portnoi, T., Roy, J. & Stark, H. (2025). *BioRxiv*.

Team, B. A. A. S., Chen, X., Zhang, Y., Lu, C., Ma, W., Guan, J., Gong, C., Yang, J., Zhang, H. & Zhang, K. (2025). *BioRxiv*, 2025.2001. 2008.631967.
